# Screening for Occult Cancer in Patients with Venous Thromboembolism

**DOI:** 10.3390/jcm9082389

**Published:** 2020-07-27

**Authors:** Julien D’Astous, Marc Carrier

**Affiliations:** 1 Department of Medicine, Centre Hospitalier de l’Université de Montréal, Université de Montréal, 1000 Saint-Denis Street, Montréal, QC H2X 0C1, Canada; jdastous@toh.ca; 2 Division of Hematology, Department of Medicine, Ottawa Hospital Research Institute, General Campus, University of Ottawa, 501 Smyth Road, Box 201A, Ottawa, ON K1H 8L6, Canada

**Keywords:** venous thromboembolism, venous thrombosis, neoplasia, occult cancer

## Abstract

Unprovoked venous thromboembolism (VTE) can be the first sign of an occult cancer. The rate of occult cancer detection within 12 months of a newly diagnosed unprovoked VTE is approximately 5%. Therefore, it is appealing for clinicians to screen patients with unprovoked VTE for occult cancer, as it could potentially decrease cancer-related mortality and morbidity and improve quality of life. However, several randomized controlled trials have failed to report that an extensive occult cancer screening strategy (e.g., computed tomography of the abdomen/pelvis) is improving these patient-important outcomes. Therefore, clinical guidance documents suggest that patients should only undergo a limited screening strategy including a thorough medical history, physical examination, basic laboratory investigations (i.e., complete blood count and liver function tests), chest X-ray, as well as age- and gender-specific cancer screening (breast, cervical, colon and prostate). More intensive occult cancer screening including additional investigations is not routinely recommended. This narrative review will focus on the epidemiology, timing, and evidence regarding occult cancer detection in patients with unprovoked VTE.

## 1. Introduction

The association between venous thromboembolism (VTE) and potential occult cancer is well established. Patients with newly diagnosed VTE are at increased risk of occult cancer diagnosis within the first 6 to 12 months following the thrombotic event. However, the extent to which clinicians need to screen patients for occult cancer at the time of VTE diagnosis, and its potential benefits on cancer-related morbidity and mortality, are unclear. This review focuses on the epidemiology, risk factors, timing, and effectiveness of screening strategies for occult cancer detection among patients with unprovoked VTE. The review will also summarize the evidence regarding the use of extensive occult cancer screening strategies in patients with unusual sites thrombosis.

We will use two patient-cases to highlight important knowledge gaps and apply the available evidence to provide some clinical recommendations for the clinical scenario. The aim of this narrative review is to revisit the evidence regarding occult cancer screening in patients with acute unprovoked VTE. We aim to critically appraise the current International Society of Thrombosis and Haemostasis (ISTH) guidance documents and understand how the current evidence translated into these recommendations. We also aim on assessing potential risk factors associated with occult cancer in patients with unprovoked VTE. This is highly relevant, as future trials are ongoing and are now focusing on high-risk patients. Finally, this review assesses the current knowledge and provides a framework for future evidence using the most recent high-quality evidence (i.e., randomized controlled trials) when available.

## 2. Epidemiology of Cancer in VTE and General Population

Several studies have confirmed the increased risk of occult cancer detection in patients with VTE [1,2]. Data from a Swedish registry assessing the incidence of cancer in patients with suspected deep vein thrombosis (DVT) reported a significant increase in observed cases of cancer diagnosis in patients with DVT compared to those in whom DVT had been ruled out [1]. More recent data from a large case-control study of elderly adults in the United States showed that VTE was associated with a 15% increased risk of any cancer (Odds Ratio (OR) = 1.15; 95% CI, 1.11–1.20) [2].

## 3. Epidemiology of Occult Cancer after VTE and Risk Factors

The incidence of occult cancer in patients with VTE has been the subject of numerous publications. In 1992, Prandoni et al. reported an incidence of cancer of 5.2% over a two-year follow up in 250 patients with DVT [3]. A Swedish registry analysis reported that 11% of patients with provoked and unprovoked DVT subsequently developed cancer [1]. A more recent systematic review and meta-analysis reported a prevalence of occult cancer of 6.3% (95% CI, 5.6% to 6.9%) within 12 months from VTE diagnosis [4].

The incidence of occult cancer detection varies according to the presence or absence of provoking factors for VTE. Only 1.9% of patients with provoked VTE have been reported to develop cancer during follow-up compared to 7.6% in patients with unprovoked events [3]. A systematic review also reported 12-month prevalence of occult cancer to be 10% (95% CI, 8.6% to 11.3%) and 2.6% (95% CI, 1.6% to 3.6%) in patients with unprovoked and provoked VTE, respectively [4]. A recently published update of this systematic review and individual patient-level meta-analysis (IPDMA) reported a lower 12-month prevalence of occult cancer detection of 5.2% (95% CI, 4.1% to 6.5%) in patients with unprovoked VTE [5].

The incidence of occult cancer detection also varies according to the presence or absence of provoking factors for VTE and if the VTE is an initial or a recurrent event. Patients with recurrent VTE seem to be at a particular higher risk (OR 4.3; 95% CI, 1.2 to 15.3) compared to patients having a first unprovoked event [3].

Risk factors associated with an increased risk of occult cancer in patients with unprovoked VTE have also been described. A post-hoc analysis of a randomized controlled trial of patients with an unprovoked VTE comparing a limited to a more extensive occult cancer screening strategy reported that age, previously provoked VTE, and current smoking were associated with a higher hazard of having a diagnosis of cancer in the year following VTE diagnosis [6]. Similarly, a post-hoc analysis of a randomized controlled trial comparing a limited screening strategy with a screening strategy based on ^18^F-fluorodeoxyglucose PET/CT for detection of occult cancer in patients with unprovoked VTE reported that an age over 50 years old was associated with occult cancer detection. Other potential risk factors reported in this analysis were male gender, leukocytosis, and thrombocytosis [7]. A risk score for occult cancer detection in patients with VTE was developed from a nested case-control study of patients included in the RIETE (Registro Informatizado Enfermedad ThromboEmbólica) registry. Factors independently associated with an increased risk of occult cancer detection included: older age (>70 years old), chronic lung disease, thrombocytosis, and anemia [8]. However, this risk score has not yet been externally validated. A systematic review and IPDMA demonstrated that the 12-month prevalence of occult cancer is 6.8% (CI, 5.6% to 8.3%) and 1% (CI, 0.5% to 2.3%) in patients older or younger than 50 years, respectively (OR, 7.1; CI, 3.1–16, *p* < 0.001) [5]. Conversely, the use of estrogen seems to be associated with a lower 12-month prevalence of cancer when compared to women not receiving estrogen [5,8]. When considering gender as a risk factor for occult cancer, available evidence is conflicting. Although men with an unprovoked VTE were found to have a higher risk of occult cancer detection (3.8% vs. 8.7%) in a prior study, the systematic review and IPDMA, reported a 12-month prevalence that was similar for women and men at 5% (CI, 3.4% to 7.5%) and 5.7% (CI, 3.8% to 8.5%), respectively [5,7]. Similarly, there was a similar rate of men with or without occult cancer in another study comparing a limited to a more extensive occult cancer screening strategy (67.6% and 63.6%, respectively) [6]. Therefore, age seems to be the only risk factor consistently associated with an increased risk of occult cancer.

Studies have also evaluated if the site and extent of index VTE was a risk factor for occult cancer detection. The 12-month prevalence of cancer according to the index VTE were similar in patients with pulmonary embolism (PE) (with or without DVT) and in patients with DVT only (5.2% (CI, 3.2% to 8.2%) and 5.6% (CI, 4.4% to 7.2%) respectively). The severity of the index event is not reported [5]. Data from the RIETE registry showed that in patients with unprovoked VTE who develop cancer during the follow-up period, 32% had PE, 49% had DVT, and 19% had concomitant DVT and PE. The severity of the index event is not reported, but it is reported that 84% of patients had a proximal DVT [8]. None of the reviewed studies commented on the severity of the index VTE as a risk factor for occult cancer. Table 1 outlines the suggested risk factor with odds ratio or hazard ratio found in the previous studies.

## 4. Timing of Cancer Diagnosis after VTE

The risk of occult cancer detection is higher in the first year after a VTE. In 1994, Nordstrom et al. reported that for all cancers, the standardized morbidity ratio in the first 6 months was 5.3 (95% CI, 4.1–6.7) and 2.1 (95% CI, 1.5–2.9) for patients with and without DVT, respectively [1]. After 6 months, this ratio was no longer statistically different between the two groups [1]. Similar results were reported in a retrospective cohort study using a Danish registry. The risk of cancer for patients with unprovoked DVT and PE was three times higher than the expected risk in the first 6 months, after which it sharply declined [9]. A case control study also assessed the risk of cancer in 1495 patients with VTE following the initial 6 months of follow up. Patients were followed for an additional 30 months. The hazard ratio for new cancer diagnosis in patients with VTE compared to patient without VTE was 1.09 (95% CI, 0.59–1.34). Similar results were reported if analysis was restricted to unprovoked VTE [10].

## 5. Evidence Related to Screening for Occult Cancer

Occult cancer screening at the diagnosis of VTE is appealing to clinicians, as it could be considered in order to potentially diagnose cancer at earlier stages and hopefully reduce cancer-related morbidity and mortality. However, the adverse effects of occult cancer screening should also be considered. Invasive testing can be costly and has the potential for major complications and induces anxiety to patients.

In 2004, the Extensive Screening for Occult Malignant Disease in Idiopathic Venous Thromboembolism (SOMIT) trial, a multicenter randomized clinical trial assessing 2 strategies (extensive diagnostic screening or no further diagnostic studies) was published [11]. Patients with a first unprovoked symptomatic DVT or PE underwent a routine cancer screening. This routine screening included a clinical history, physical examination, basic laboratory tests (complete blood count (CBC), AST/ALT, ALP, calcium, and urinalysis), and chest radiography. Patients were then randomized according to a Zelen design to an extensive diagnostic screening or no further testing. Patients in the extensive screening group underwent an ultrasound of abdomen and pelvis, CT of abdomen and pelvis, lower and upper endoscopies (or barium study), hemoccult, sputum cytology, and tumor markers. Women also received a mammography and Pap smear; men also received a transabdominal ultrasound of the prostate and prostate-specific antigen (PSA) measurement. These tests needed to be completed within a four-week period after the diagnosis of VTE. Further testing was done if there was a suggestion of cancer based on the previous investigation. Screening tests were not repeated during the study period. The primary outcome of the SOMIT trial was cancer-related mortality. The follow-up period was 24 months. The trial included 201 patients, 99 were allocated to the extensive screening group and 102 to the control group. A total of 32 patients were initially excluded because cancer was diagnosed on initial assessment (routine screening before randomization). In 13.1% of the extensive screening group, this extensive work-up resulted in a confirmed diagnosis of cancer. In the extensive screening group, 1% developed a cancer during follow up compared to 9.8% in the control group (risk difference of 8.8%; 95% CI, 0.8% to 19.1%). Therefore, of 14 cancers in the extensive screening group, 13 of them were diagnosed at the time of extensive screening. The primary outcome (cancer-related mortality) occurred in 2% and 3.9% of the patients in the extensive group and in the control group, respectively (absolute difference: 1.9% (95% CI, −5.5% to 10.9%)). Despite not reaching a statistically significant difference in cancer-related mortality, this study showed that an extensive occult cancer strategy might be beneficial and suggested that CT of abdomen and pelvis may have the highest yield if extensive screening is considered [11].

The Screening for Occult Cancer in Unprovoked Venous Thromboembolism (SOME) trial was a multicenter, open-label, randomized clinical trial comparing comprehensive CT of the abdomen and pelvis in addition to limited occult cancer screening with limited occult cancer screening alone in patients with a first unprovoked VTE [12]. Patients in the limited screening group underwent a clinical history, physical examination, laboratory tests (CBC, serum electrolyte and creatinine levels and liver-function testing), chest radiography, and gender-specific screening if it had not been performed in the previous year. Age and gender-specific screening included a breast examination, mammography, or both and a Pap smear and a pelvic examination in women; whereas men underwent a prostate examination, PSA, or both. The comprehensive CT group also underwent a CT of the abdomen and pelvis (with virtual colonoscopy and gastroscopy, enhanced CT of the liver, parenchymal pancreatography, and enhanced CT of the distended bladder). The different occult cancer strategies (including CT) had to be completed within six weeks of randomization. The primary outcome was newly diagnosed cancer during the follow-up period in patients with a negative screening result initially. The follow-up period was one year. A total of 862 patients were randomized, of which 854 patients were included in the intention-to-test analysis. During the one-year follow up, 14 patients (3.2%; 95% CI, 1.9% to 5.4%) and 19 patients (4.5%; 95% CI, 2.9% to 6.9%) received a diagnosis of cancer in the limited-screening and limited-screening-plus CT groups, respectively. When comparing missed cancer (cancer diagnosed during follow up after a negative screening results), it was found that 4 of 14 cancers were missed by the limited screening strategy, while 5 of 19 cancers were missed by the limited-screening-plus CT (*p* = 1.0). It is important to note that the prevalence of cancer was lower than expected (3.9%; 95% CI, 2.8% to 5.4%). This might be related to the inclusion of younger patients (mean age 53.7 and 53.4 in the limited screening and limited screening plus CT, respectively) [12].

A potential attractive strategy for occult cancer screening in patients with unprovoked VTE could be the use of ^18^F-fluorodeoxyglucose (^18^F-FDG) PET/CT. It would allow using a single non-invasive test instead of multiple different tests as used in the previous trials. An open-label, multicenter, randomized controlled trial compared a limited screening strategy and a strategy combining limited screening and ^18^F-FDG PET/CT in patients with unprovoked VTE (Limited Screening With Versus Without ^18^F-fluorodeoxyglucose PET/CT for Occult Malignancy in Unprovoked Venous Thromboembolism (MVTEP)) [13]. The limited screening strategy included a clinical history, physical examination, laboratory tests (CBC, C-reactive protein, ALT/AST, ALP and calcium), chest radiography, and age and gender-specific cancer screening test (PSA in men older than 50 years; mammography in women older than 50 years and Pap smear in all women). The primary outcome was the proportion of patients receiving a cancer diagnosis after the completion of the initial screening strategy for which they were randomized. Follow up was planned for a total of 24 months. A total of 200 patients were allocated to the ^18^F-FDG PET/CT group and 199 to the limited screening group. Cancer was diagnosed in 5.6% and 2% in patients of the ^18^F-FDG PET/CT and limited screening group, respectively (Absolute risk difference: 3.6%; 95% CI, −0.4% to 7.8%). Additional testing because of initial positive tests were required in 23.7% of patients in the ^18^F-FDG PET/CT group versus 16.2% in the limited screening group. During follow up, one patient in the ^18^F-FDG PET/CT group was diagnosed with cancer compared to nine patients in the limited screening group. Accordingly, the incidence of occult cancer was 0.5% in patients with a negative initial ^18^F-FDG PET/CT, whereas it was 4.7% in the patients in the limited screening group (Absolute risk difference: 4.1%; 95% CI, 0.8% to 8.4%). Despite the negative results of this study, it is interesting to emphasize the significant reduction in cancer diagnosis during follow up in the ^18^F-FDG PET/CT group. The potential limitation of this strategy includes the need for further potentially invasive procedures in the ^18^F-FDG PET/CT group because of false-positive results. The frequency and invasiveness of additional testing following those strategies were assessed in a post-hoc analysis of the MVTEP study. A total of 59 (22.8%) additional diagnostic procedures were performed in patients in the ^18^F-FDG PET/CT group compared to 53 (16.2%) additional tests in the limited screening group (Absolute risk difference: 6.6%; 95% CI, −1.3% to 14.4%, *p* = 0.13). However, more patients in the ^18^F-FDG PET/CT group underwent invasive testing when compared to the limited screening group (Absolute risk difference 5.1%; 95% CI, 0.5% to 10%, *p* = 0.03). No procedure-related complications were reported [14].

## 6. Future Directions

Other trials are on-going and will help provide additional insight and further evidence on occult cancer screening. The MVTEP/SOME 2 trial is a French-Canadian collaboration randomizing older patients (≥50 years) with unprovoked VTE to a limited screening strategy along or in combination with PET-CT (NCT04304651). Similarly, another multicenter, randomized controlled trial is comparing a limited screening strategy to an extended screening strategy (using a PET-CT) in patients deemed at high risk of cancer according to the previously mentioned risk score developed from the RIETE registry [8] (NCT03907583). Other means of occult cancer detection are also being investigated. Platelets have an effect on tumor cells by promoting progression and spread of cancer. Platelets may also have the ability to interact with tumor cells in a process called “education”. During this process, platelets acquire tumor-associated biomolecules. This transfer of biomolecules and external stimuli change the RNA in circulating platelets creating tumor-educated platelets (TEPs). Therefore, circulating platelets carry an index of RNA that can help in diagnosing cancer [15]. Further studies showed that a RNA profile from TEPs was able to identify patients with cancer when compared to cancer-free patient with an accuracy ranging from 84% to 96%. TEPs were also able to localize the primary tumor with an accuracy of 71% [16]. This platelets RNA profiling is being evaluated in a prospective cohort study to detect occult cancer in patients with unprovoked VTE (NCT02739867).

## 7. Clinical Practice Guidelines and Guidance Documents

The scientific subcommittee of the International Society on Thrombosis and Haemostasis has recently provided guidance on this clinical challenge (Table 2) [17]. Patients with an unprovoked VTE should undergo a limited cancer screening and age- and gender-specific cancer screening. A limited cancer screening includes a clinical history, physical examination, laboratory tests (CBC, calcium, urinalysis, and liver function tests), and chest radiography. Age- and gender-specific cancer screening (i.e., breast, cervix, colon, and prostate cancer) should follow national recommendations. Similar recommendations are applicable for patients with recurrent unprovoked VTE, but a lower threshold for cancer detection may be reasonable. Routine cancer screening is not recommended for patients with a provoked VTE [17]. Clinical trials investigating whether extensive occult cancer screening strategies including additional diagnostic imaging is beneficial in high-risk patients are ongoing. PET/CT is being evaluated for occult cancer detection in patients older than 50 years and in patients deemed at high-risk according to risk factors. In the future, we might be able to tailor occult cancer screening on the basis of the associated underlying risk of occult cancer in patients with unprovoked VTE. However, this strategy needs to be investigated before being used in clinical practice. On the basis of current available evidence, we cannot provide an evidence-based recommendation on extensive occult cancer screening for these specific patient groups.

Two cases are presented to illustrate the principles discussed above:

**Case 1**

A 67 year-old male was referred to the Thrombosis clinic for assessment of an unprovoked proximal DVT. His past medical history is significant for hypertension, dyslipidemia, and benign prostate hyperplasia. He denied any past history of VTE, stroke and myocardial infarction. He is a former smoker (stopped seven years ago) with a history of 45 packs per year.

He presented to the emergency department with a two-day history of right leg pain and swelling. He did not have any respiratory symptoms. There were no provoking factors. Vital signs were within normal limits. Right leg ultrasonography revealed a common femoral vein thrombus. He was initiated on rivaroxaban 15 mg PO twice daily and referred to the Thrombosis clinic.

The patient underwent colon and prostate cancer screening within the last five years and one year, respectively. The patient asked if other tests are required as he is concerned about the association between VTE and cancer (Table 2).

On further assessment, the patient did not report other symptoms and there were no concerns on physical examination. Basic laboratory tests done at the emergency room including a CBC, creatinine, electrolytes (including calcium), and liver function tests were within normal limits. It was suggested that his family physician continue age-specific cancer screening. A chest radiography and urinalysis was also suggested to complete initial cancer screening.

**Case 2**

A 55-year-old previously healthy woman presented to the emergency department because of right flank pain. Vital signs were within normal limits. Initial blood work was normal (CBC, renal function, and liver function test). CT of the abdomen revealed a complete occlusion of the main portal vein consistent with splanchnic vein thrombosis (SVT). She was seen by a gastroenterology specialist who will follow her as an outpatient. The patient was initiated on therapeutic doses of low-molecular-weight-heparin (dalteparin 200 units/kg sc daily) and referred to the Thrombosis clinic by gastroenterology to investigate potential causative factors.

## 8. Epidemiology

The most common sites of VTE are the deep veins of the lower extremities and the pulmonary arteries. However, VTE can happen in any vein. Unusual site thrombosis including SVT, cerebral vein thrombosis (CVT), upper extremity (UE) DVT, and retinal vein occlusion (RVO) among other sites [18]. Splanchnic vein thrombosis encompasses hepatic vein thrombosis (Budd-Chiari syndrome), portal vein thrombosis, mesenteric vein thrombosis, and splenic vein thrombosis. Previous older studies might have underestimated its prevalence. A hospital registry reported an incidence of SVT of less than four cases per million people [19], but a population-based study including more than 20,000 autopsies reported a prevalence of 1% [20]. A more recent publication reported the prevalence of incidental SVT in patients undergoing abdominal CT. It found an estimated prevalence of 1.74% (95% CI, 1.29% to 2.34%) [21].

## 9. Risk Factors and Causes

Causes of SVT include cancer, cirrhosis, abdominal infection, pancreatitis, inflammatory bowel diseases, abdominal surgery, trauma, inherited thrombophilia, acquired thrombophilia, oral contraceptives or hormone replacement therapy, and pregnancy. The presence of multiple causative factors is often found [22]. Of those, undiagnosed myeloproliferative neoplasm is of interest as it can be detected by testing for the JAK2V617F mutation.

The association of JAK2V617F mutation and VTE was reviewed in a systematic review and meta-analysis published in 2009 [23]. Out of the 3508 included patients, 831 had a SVT and of those, 280 were found to have the JAK2V617F mutation (mean prevalence of 32.7%; 95% CI, 25.5% to 35.9%). This prevalence was even higher in patients with unprovoked SVT. Case-control studies also reported that the JAK2 mutation was significantly more likely to be present in patients with SVT than controls (OR 53.98; 95% CI, 13.1–222.45). It is also interesting to note that 52.4% (95% CI, 38% to 66.5%) of patients with JAK2 mutation did not have a diagnosis of myeloproliferative neoplasm at presentation of SVT but were diagnosed during the follow up [23]. Prevalence of JAK2 mutation was also reported to be 0.88% (95% CI, 0.44% to 1.45%), 2.57% (95% CI, 0.97% to 4.91%) and 0.99% (95% CI, 0.05% to 3.2%) in patients with DVT/PE, CVT and RVO, respectively [23].

## 10. Clinical Practice Guidelines and Guidance Documents

The recent guidance from the scientific subcommittee of the International Society on Thrombosis and Haemostasis also commented on cancer screening in patients with VTE at unusual sites (Table 2) [17]. Further imaging tests are not recommended for SVT because the CT abdomen and pelvis is often used for the initial diagnosis and would be adequate for occult cancer detection. However, given the higher prevalence of JAK2 mutation in patients with SVT, JAK2V617F mutation testing to screen for a myeloproliferative disorder should be considered in patients with unprovoked events [17].

## 11. Back to Case 2

On review of the CT abdomen, no intra-abdominal malignancy was detected. No other causative factors were found on the questionnaire and physical examination. The patient had recent age and gender-specific cancer screening. The screening for JAK2V617F mutation was positive. The patient did not have other findings suggestive of a myeloproliferative disorder. She was referred to hematology for further evaluation and management.

## Figures and Tables

**Table 1:** Suggested risk factors ^1^.

Risk Factors	OR/HR (95% CI)	Risk Factors (Continued)	OR
Patient Characteristics		Index Event	
Age older than 50 years	OR 7.1 (3.1–16) [5]	Unprovoked VTE	OR 1.42 (1.15–1.75) [8]
Age older than 60 years	HR 3.11 (1.41–6.89) [6]		
Age older than 70 years	OR 1.93 (1.58–2.35) [8]		
Male gender	OR 1.3 (1.07–1.58) [8]	Laboratory tests	
Use of estrogen	OR 0.29 (0.14–0.58) [8]	Anemia	OR 1.66 (1.35–2.03) [8]
Comorbidities		Thrombocytosis	OR 1.36 (1.01–1.83) [8]
Chronic lung disease	OR 1.71 (1.31–2.23) [8]		
Active smoker	HR 2.8 (1.24–6.33) [6]		

OR: odds ratio; HR: hazard ratio. ^1^ From post-hoc analyses of randomized control trial and a nested case-control study of patients included in the Registro Informatizado Enfermedad ThromboEmbólica (RIETE) registry.

**Table 2:** Recommendations.

	Limited Screening ^1^	Age- and Gender-Appropriate ^2^	Other
Provoked VTE	Not recommended	Not recommended	None
Unprovoked VTE	Recommended	Recommended	None
Recurrent unprovoked VTE	Recommended	Recommended	Maintain a low threshold for further investigation ^3^
VTE at unusual sites	Recommended	Recommended	Search for JAK2V617F mutation

^1^ Clinical history, physical examination, laboratory tests (complete blood count, calcium, urinalysis, and liver function tests) and chest radiography. ^2^ Breast, cervix, colon, and prostate cancer screening according to local national guidelines. ^3^ No definitive suggestion in term of investigation or alarming signs.
